# Associations between sexual violence and women's sexual self-consciousness

**DOI:** 10.4314/ahs.v23i4.42

**Published:** 2023-12

**Authors:** Ghaffarian Jafarzade Sara, Bostani khalesi Zahra, Niknami Maryam, Maroufizadeh Saman

**Affiliations:** 1 Department of Midwifery, School of Nursing and Midwifery, Guilan University of Medical Sciences, Rasht, Iran; 2 Department of Biostatistics, School of Health, Guilan University of Medical Sciences, Rasht, Iran

**Keywords:** Women of reproductive age, sexual violence, sexual self-consciousness

## Abstract

**Background:**

Sexual violence is a global public health problem that has serious and multiple consequences for the victims' health.

**Objective:**

This study was conducted to determine associations between sexual violence and women's sexual self-consciousness.

**Methods:**

In the analytical cross-sectional study, 340 eligible married women of reproductive age who have been referred to comprehensive health centers of Rasht, participated. Simple random sampling followed by cluster sampling was used to reach eligible study participants. The data collection tool was a three-part questionnaire including a demographic information form, sexual violence questionnaire, and sexual self-consciousness scale.

**Results:**

More than 66% of the participants in this study were exposed to sexual violence by their current spouses/partners. The highest prevalence of sexual violence was in the dimension of unwillingness to have sex (49%), and the lowest in the verbal dimension was 2.1%. Participants were 4.11 ± 5.18. There was a significant positive correlation between the total score of sexual violence and the total score of sexual self-consciousness of participants (P <0.001, r = 469).

**Conclusion:**

According to the findings, there is a significant positive correlation between SSC and sexual violence, so prevention policies for sexual violence should be focused on skill-based programs and empowering women.

## Introduction

Sexual violence includes unwanted penetration, and an array of types of coercion, from social pressure and intimidation to physical force to verbal harassment^1^. Therefore, four characteristics of sexual violence can be listed: dissatisfaction, complete action or attempt to do so, type of force (for example, physical or non-physical), and type of sexual activity, from non-contact sexual harassment to sexual penetration^2^. Sexual violence is a worldwide phenomenon and the most common example of violence against women. One in three women is sexually abused by her husband^3^. The prevalence of sexual violence in developing countries is between 68-18%^4^ in developed countries 28%^3^ and in Asian countries such as Pakistan^5^ and Turkey^6^ 4.53% and 3.6%, respectively. Studies in Iran have reported a high rate of sexual violence (18 to 55%)^3^,^7^.

Victims of sexual violence by their spouse suffer psychological damage long after experiencing violence^8^. According to research, victims of sexual violence by spouses show more severe post-traumatic reactions than those who have been abused by strangers or those who have simply experienced physical violence from their spouses^9^. Women who experienced sexual violence from their husbands are more likely to develop post-traumatic stress disorder (PTSD) and also to commit suicide^10^. Many women who are sexually abused by their husband's experience depression, anxiety, fear, low self-esteem, guilt, and sleep disorders too^11^. Spousal violence against women can have deadly consequences, such as murder or suicide^12^. Sexual violence by a partner can lead to unwanted pregnancies, abortions, and sexually transmitted infections, including HIV^13^. Women who have been physically or sexually abused are 1.5 times more likely to be infected with a sexually transmitted infection and to be HIV-positive in some areas than women who have not experienced sexual violence by their husbands^14^. Abortion is also twice as likely to happen to them^9^. Sexual violence is an example of the sexual behaviour of individuals and self-consciousness can improve sexual behavior^10^. In addition, sexual self-consciousness (SSC) predicts sexual function^15^ and indicates sexual thoughts, attitudes, actions, and feelings of individuals^16^. SSC consists of the tendency to think and reflect on the sexual life perspective, the nature of sexuality, and feelings about the social pressure regarding sexual behaviors^17^. In other words, SSC means knowledge and consciousness of a person about his gender and sexual issues^18^. SSC is one of the most important aspects of life skills that enables people to identify all of their traits, each of which can affect their sexual satisfaction in some way^19^. Researchers emphasize the important role of SSC in marital satisfaction and improving sexual health^20^. Low sexual consciousness is one of the main reasons for the increase in AIDS, sexually transmitted diseases, unwanted pregnancies, abortions, and marital dissatisfaction^14^. On the other hand, misconceptions and insufficient consciousness of desirable sexual behaviours also can lead to dissatisfaction and sexual violence by the spouse 3.

Determining sexual violence rates and related factors can be helpful measures to be taken to decrease sexual violence, social awareness programs to be organized, and all kinds of strategies, and new regulations to be developed. In this sense, it is considered that it is important to determine the relationship between sexual violence and SSC, which defines the sexual behaviour and characteristics of an individual, as well as sexual attitude, as shaped by social roles. therefore, this study was conducted to determine the determine associations between sexual violence and women's sexual SSC.

## Method

In the analytical cross-sectional study, 340 eligible married women of reproductive age who have been referred to comprehensive health centers of Rasht, participated. Simple random sampling followed by cluster sampling was used to reach eligible study participants. Inclusion criteria included: consent to participate in the study, having a spouse and sex, no disease (cardiovascular disease, epilepsy) Diabetes, high blood pressure (no use of drugs related to mental illness), self-reported lack of severe psychological stress such as an accident or loss of first-degree family members during the last 3 months. The exclusion criteria were the failure to fully answer the standard questionnaire.

The sample of the study was calculated by the G * Power program as 340 women with a 1-point deviation and 90% strength from the known average SSC obtained from the study of Alan Dikmen & Cankaya 21. It was decided to increase the calculated sample size (340 women) by 10% considering the probable non-responses, which made the sample size 374.

Data were collected using a demographic information form and two scales, sexual violence questionnaire (SVQ), and sexual self-consciousness scale (SSCS).

A self-report questionnaire with socio-demographic data (age, education, occupation, age of spouse, education of spouse, occupation of spouse, place of residence, marital status, number of children, marriage history, economic status, smoking and drug use by spouse, insurance status, housing, age of first sexual activity, The frequency of sexual intercourse during the week, sexual abuse in childhood). The demographic information form for the face validity review was given to 10 faculty members and after applying the point of view, the final correction was made to it.

The SVQ is a standard 16-item tool in 4 dimensions (verbal violence, reluctance to have sex, coercion not to use contraception, and physical harm during intercourse) designed and psychometric by Khalifeh et al 22. How to score it on a 5-point Likert scale (never, rarely, sometimes, often, always) and is scored and calculated from 0 - 4. The average of the total scores related to the question-naire items shows the overall score of sexual violence. To determine the validity of the sexual violence questionnaire, quantitative content validity based on content validity ratio indices and content validity index based on the opinions of a ten-member panel consisting of university faculty members were used.

The SSCS has 12 items and was designed by van Lankveld et al 23. This questionnaire has two subscales of sexual self-focus and sexual shame. It is scored on a Likert scale with 5 options from strongly disagree (score 0) to strongly agree (score 4). The SSC total score range is 0-48 and in each of the subscales of sexual self-focus and sexual shame is 0-24. A higher score indicates greater SSC. van Lankveld et al. confirmed the content validity of this questionnaire and reported the total alpha coefficient of the questionnaire as 85. Tabatabayi et al 24 confirming the content validity, reported a total Cronbach's alpha coefficient of 77.

The study was approved by the ethical committee of Guilan University of Medical Sciences (project number is IR.GUMS.REC.1400.365). Participation in this study was voluntary. Women who agreed to participate in the study completed the written consent form. Participants could withdraw from the study at any time they wanted, and participants were assured that their information would be kept confidential.

In this study, the values of qualitative variables are shown as (the percentage of frequency), and the values of quantitative variables are shown as the standard deviation of the mean. Sexual violence scores and SSC relationships were assessed using the Spearman correlation coefficient. Simple (and multivariate) multiple logistic regression analyses were used to investigate the factors related to sexual violence. The results of these two analyses were presented as raw odds ratio (OR) and adjusted odds ratio (aOR) with 95% confidence intervals (95% CI). Data were analysed using SPSS software version 16 and the significance level was considered 0.05.

## Results

The mean age of participants was 35.73± 8.91 years. About 42% of the participants had a university education, 85.4% were housewives and 86.9% were city dwellers. 2.7% of marriages were temporary, 8.1% of them were remarried and 8.2% had no children. The mean age of the spouses was 39.90 ± 9.53 years. 39.1% of the spouses had a university education, 47.8% were self-employed, and 31.6% were smoking. About 67.1% of the participants had personal houses, and 78.8% had insurance. The mean age of participants' first sexual activity was 21.5 ± 91.11 years and 47.5% of them had sexual activity 2 to 3 times a week. 9.3% of the participants reported a history of child sexual abuse. Overall, 66.3% of participants reported experiencing sexual violence by their spouses. The mean total score of participants' sexual violence was 4.11± 5.18. The prevalence of sexual violence in verbal dimensions was 2.1%, reluctance to have sex was 49%, spouse humiliation during sexual intercourse was 46%, coercion not to use contraceptive methods was 17% and physical harm was 17.9%. The highest prevalence in terms of the type of sexual injury was related to genital pain, infection in the genital area, and injury to the genital area, respectively (Table 1).

**Table 1:** Frequency distribution of the type of injury caused by forced sex in participants

Type of injury	frequent				
	Never	rarely	Sometimes	often	Always
	Frequency (percentage)				
Genital bleeding	291 (86.9)	30(9.0)	8 (2.4)	6(1.8)	0(0)
Genital pain	176(52.5)	63(18.8)	53(15.8)	25(7.5)	18(5.4)
Infection in the genital area	228(68.1)	48(14.3)	38(11.3)	16(4.8)	5(1.5)
Injury to the genital area	294(97.8)	28(8.4)	5(1.5)	3(0.9)	5(1.5)

The total SSC score of the study participants was 18.43 ± 7.78, and the SSC score in the field of sexual shame was 6.71±5.93 and in the area was 11.72±5.18. There was a significant positive correlation between the total SV score and the SSC score of the participants (P <0.001, r = 469); in other words, by increasing SSC score in participants, their SV score will rise. There is also a significant positive correlation observed between the SV score of and the scc score in the area of sexual shame of the participants (P <0.001, r = 0.369 (Table 2).

**Table 2:** Associations between sexual violence and women's sexual self-consciousness

Subscales of sexual self-consciousness	The total score of sexual violence	
	Spearman correlation coefficient	*P*
Sexual shame	0.469	<0.001
Sexual self-focus	-0.026	0.635
The total score of sexual self-consciousness	0.368	<0.001

### Correlation analysis

#### Univariate Logistic regression analysis

In univariate analyzes, the chance of sexual violence in women with education less than a diploma was 2.61 times higher than in women with a university degree (1.42 - 4.80: 95% CI, OR = 2.61). Those who had three or more children were 55% higher than women who had one or two children (CI: 0.22 - 0.92 - 95%, OR = 0.45). The chance of sexual violence among women whose husbands had diplomas was 67 times higher than those whose husbands had a university degree (0.98–2.86: 95% CI, OR = 1.67). Compared to women whose husbands were employees, the chance of sexual violence in women with working wives (1.41 - 4.41: 95% CI, OR = 2.33 (as well as women with self-employed spouses) 3.29 - 3.87 1: 95% CI, OR = 2.24 (as more). The chance of sexual violence in women whose economic status was less than adequate is 2.02 equal to women whose economic status was adequate) 4.01 - 1.01: 95% CI, OR = 2.02. The chance of sexual violence in women whose husbands smoked was 2.38 times higher than in women whose husbands did not smoke. Women who were insured were 68 percent more likely to have sexual violence than women who were not insured (3.04 - 0.93: 95% CI, OR = 1.68). The chance of sexual violence in women whose age of first sexual activity was less than 18 years was 2.7 times higher than in women whose age of first sexual activity was more than 25 years (1.40 - 5.40: 95% CI, 2.70 = OR). The chance of sexual violence in women who reported a history of child sexual abuse was 5.37 times higher than in women who did not report a history of sexual abuse in childhood (95.06 CI: 1.18 - 95%, OR = 5.37 With increasing sexual self-consciousness score, the chance of sexual violence also increased (1.14 - 1.06: 95% CI, OR = 1.10) so that for one unit increase in female sexual self-consciousness score, the chance of sexual violence increased. They increased by 10% (Table 3).

**Table 3:** Univariate and multivariate logistic regression models of sexual violence in terms of sexual self- consciousness and related factors

Variable	Category	Sexual violence	Univariate analysis		Multivariate analysis	
		Frequency(percentage)	OR (95% CI)	*P*	OR (95% CI)	*P*
Age (years)	Less than 30 years	61(61.0)	1		1	
	31-40	91(65.9)	1.24(0.73-2.11)	0.434	1.68(0.74-3.78)	0.214
	Older than 40 years	69(71.1)	1.58(0.87-2.86)	0.134	1.84(0.62-5.48)	0.273
Education level	High school	67(77.9)	2.61(1.42-4.80)	0.002	1.15(0.40-3.28)	0.801
	Diploma	73(67.6)	1.54(0.92-2.61)	0.103	1.30(0.63-2.69)	0.473
	academic education	81(57.4)		1		1
Occupation	housewife	189(66.1)	1.17(0.59-2.32)	0.655	0.75(0.30-1.83)	0.524
	worker	7(77.8)	2.10(0.38-11.46)	0.391	0.59(0.08-4.40)	0.604
	Employee	25(62.5)	1		1	
Habitat	City	192(66.0)	1		1	
	Village	29(65.9)	1.00(0.51-1.95)	0.993	0.65(0.28-1.53)	0.327
Marital status	Permanent marriage	213(65.3)	1		1	
	Temporary marriage (registered or not)	8(88.9)	4.24(0.52-34.36)	0.176	2.83(0.26-30.73)	0.393
Number of marriages	Once	202(65.6)	1		1	
	Twice	19(70.4)	1.25(0.53-2.94)	0.615	0.46(0.16-1.33)	0.152
Number of children	0	22(78.6)	1.01(0.33-3.10)	0.988	1.17(0.29-4.76)	0.821
	1-2	159(62.1)	0.45(0.22-0.92)	0.029	0.82(0.33-2.03)	0.666
	> 2	40(78.4)	1		1	
Spouse age (years)	Less than 30 years	37(63.8)	1		1	
	31-40	88(64.2)	1.02(0.54-1.93)	0.953	1.36(0.55-3.37)	0.504
	More than 40	96(68.6)	1.24(0.65-2.36)	0.515	1.39(0.45-4.26)	0.565
Level of Education	Highschool	72(74.2)	2.15(1.21-3.81)	0.0009	0.95(37-2.43)	0.913
	Diploma	74(69.2)	1.67(0.98-2.86)	0.060	0.95(0.46-1.96)	0.042
	Academic education	75(57.3)	1	-	1	-
Occupation	Employee	43(51.8)	1	-	1	-
	Worker	60(71.4)	2.33(1.23-4.41)	0.010	2.42(0.97-6.01)	0.057
	Self-employed	113(70.6)	2.24(1.29-3.87)	0.004	2.17(1.03-4.56)	0.042
	Unemployed	5(62.5)	1.55(0.35-6.91)	0.565	1.22(0.20-7.61)	0.832
The economic situation	Inadequacy	9(90.0)	5.32(0.66-42.57)	0.116	12.96(0.96-174.08)	0.053
	< enough	41(77.4)	2.02(1.01-4.02)	0.046	1.30(0.55-3.12)	0.550
	Enough	171(62.9)	1	-	1	-
Smoking and alcohol consumption by the spouse	Yes	83(78.3)	2.38(1.40-4.05)	0.001	1.98(1.06-3.72)	0.033
	No	138(60.3)	1		1	
Insurance	Yes	168(63.6)	1	-	1	-
	No	53(74.6)	1.68(0.93-3.04)	0.084	1.21(0.59-2.47)	0.600
House	Private house	148(65.2)	1	-	1	-
	Rental housing	73(67.6)	1.11(0.68-1.81)	0.666	1.03(.056-1.92)	0.913
Age of first sexual activity	Less than 18	69(79.3)	2.70(1.35-5.40)	0.005	0.61(0.20-1.88)	0.392
	18-25	108(62.4)	1.17(0.67-2.04)	0.577	0.59(0.21-1.70)	0.331
	> 25	44(85.7)	1	-	1	-
The frequency of repeated sexual intercourse during the week	0-1	98(68.1)	0.71(0.30-1.70)	0.442	0.61(0.20 – 1.88)	0/392
	2-3times	99(62.3)	0.55(0.23-1.30)	0.174	0.59 (0.21 -1.70)	0.331
	4-5 times	24(75.0)	1	-	1	-
Child sexual abuse	Yes	28 (90.3)	5.37(1.60-18.6)	0.007	3.69 (0.91 – 14.94)	0.067
	No	193 (63.5)	1	-	1	-
Sexual self-consciousness		-	1.10 (1.06-1.14)	<0/001	1/08 (1/04 – 1/12)	<0/001

OR: Raw odds ratio, aOR: Adjusted odds ratio; Confidence interval

#### Multivariate logistic regression analysis

The results of multivariate analysis (adjusted) showed the chance of experiencing sexual violence in women with spouses with self-employment compared to women with employed spouses (4.53 - 1.03: 95% CI, aOR = 2.17), in women with spouses Smoker) 3.03 - 1.06: CI95%, aOR = 1.98 (and in women with higher scores of SSC) 1.12 - 1.04: CI 95%, aOR = 1.08 (higher). Chance of sexual violence in women with factory worker spouses compared to women with employee spouses (0.6-0.97: 95% CI, aOR = 2.42), in women with insufficient income level compared to women with limited income Adequacy) 0.96 - 174-08: 95% CI, aOR = 12.96 (and in women with a history of child sexual abuse) 0.94 - 0.91: 95% CI, aOR = 3.69) was higher but these differences were not statistically significant (P = 0.057, P = 0.053 and P = 0.067, respectively).

## Discussion

In this study, the rate of women exposed to sexual violence by their current husbands/partners was 66.3% (much higher than the Iranian average), 32% (and neighbouring countries such as Turkey) (12%)^6^. The dramatic increase has occurred because women are more likely to express their experiences of sexual violence than in previous years. Also, the differences in the results of the studies may be due to the different functions in the identification, definition, and questionnaire for collecting information on sexual violence. Therefore, the rate of sexual violence in similar studies may also vary.

Although, the results of the present study in terms of the prevalence of sexual violence are the same as the results of Zarei et al.'s study 7. Zarei et al. Reported a prevalence of sexual violence of 52.4%. Because sexual violence is culturally based, there is a difference in the prevalence of sexual violence^25^. Therefore, it is predictable in different societies.

The highest prevalence of sexual violence was the unwillingness to have sex (49%), and the lowest in the verbal dimension was 2.1% (also. It was in the genital area). In this study, the mean scores of SSC of women who experienced sexual violence were significantly higher. These results were in line with those of Alan Dickman & Cankaya 21. They reported in their study that women with a high SSC score were more likely to report sexual violence. The reason can be due to women's consciousness of their rights. Women with high levels of SSC are more aware of desirable sexual behaviours and dimensions of sexual violence^26^. It disturbs the couples thinking about each other and causes them to distance themselves from each other and dissatisfaction with marital relationships^18^.

However, if SSC is strengthened in couples and they share their information raises the context of marital relations and sexual issues. At that time, look at each other with the eyes of a companion and they will never harass each other in marital relations and will not use sexual violence against each other^19^.

The results of the present study show that the chances of experiencing sexual violence are higher in women whose spouses are freelance and working compared to women with employed spouses. The results for the present study were the same as the results of Zarei et al^7^ and Bilgili & Vural^27^. They reported in their study that the more appropriate the type of job, the less violence. There is in some studies, the use of alcohol by the husband was reported as a factor associated with sexual violence, which was in line with the results of our study. Alcohol consumption affects negatively anger management and cognitive functions and increases violence against women by causing behavioural disorders^28^.

Another variable related to sexual violence in logistic regression analysis is the history of sexual abuse. A history of sexual abuse weakens people's belief in their ability to perform successful sexual behaviors^3^. In one study, women exposed to sexual violence were found to have lower sexual self-efficacy^29^. Low sexual self-efficacy can lead to re-experiencing sexual violence^8^. The trauma, injury, and fear experienced by a woman with a background of sexual violence may reduce her belief that she is sexually powerful and self-sufficient, and thus her level of sexual self-efficacy may be lower^9^. Therefore, can be said that the relationship between sexual violence and sexual self-efficacy is reciprocal. Although the present study makes an important contribution to the literature on sexual violence, it has limitations. One is the study's cross-sectional, correlational design, which precludes conclusions about causal links between the studied variables.

It is therefore important to consider that SSC, may contribute to women's lower sexual violence.

Our data are based on the participants' self-reports; participants' negative attitudes to sexuality and the sexual taboos that women face issues may have affected the responsiveness of study participants. Despite these limitations, the results of this study have important implications for understanding the importance of women's SSC as well as their experience of sexual violence. These results allow healthcare providers to further their understanding of the link between sexual violence and women's SSC as markers of sexual health. Clinically, this study emphasizes the importance of considering SSC when working with women presenting with sexual difficulties and/or sexual violence. Sexual therapists should take into account SSC in their assessment and treatment plan when working to reduce women's sexual violence.

## Conclusion

Findings showed that more than 66% of the participants in this study reported sexual violence. There is a significant positive correlation between SSC and sexual violence. Since SSC is a dimension of life skills, it can be a kind of barrier to sexual violence based on the results of this study. Therefore, empowerment of women in this skill, especially SSC through education by health care providers should be considered.

Longitudinal studies are recommended to confirm the link between sexual violence and women's SSC. The sexual issue is obviously a dyadic configuration, so it will be important in future studies to collect data from both men and women.
